# Contrast-Induced Nephropathy in Patients Undergoing Percutaneous Coronary Intervention

**DOI:** 10.4061/2010/649164

**Published:** 2010-09-19

**Authors:** Sana Shoukat, Saqib A. Gowani, Asif Jafferani, Sajid H. Dhakam

**Affiliations:** ^1^Cardiology Section, Department of Medicine, The Aga Khan University, Stadium Road, P.O. Box 3500, Karachi 74800, Pakistan; ^2^MBBS Program, The Aga Khan University, B-4, Al-Qahir Apt., Violet Street, Garden East, Karachi 74550, Pakistan

## Abstract

Contrast Induced Nephropathy (CIN) is a feared complication of numerous radiological procedures that expose patients to contrast media. The most notorious of these procedures is percutaneous coronary intervention (PCI). Not only is this a leading cause of morbidity and mortality, but it also adds to increased costs in high risk patients undergoing PCI. It is thought to result from direct cytotoxicity and hemodynamic challenge to renal tissue. CIN is defined as an increase in serum creatinine by either ≥0.5 mg/dL or by ≥25% from baseline within the first 2-3 days after contrast administration, after other causes of renal impairment have been excluded. The incidence is considerably higher in diabetics, elderly and patients with pre-existing renal disease when compared to the general population. The nephrotoxic potential of various contrast agents must be evaluated completely, with prevention as the mainstay of focus as no effective treatment exists. The purpose of this article is to examine the pathophysiology, risk factors, and clinical course of CIN, as well as the most recent studies dealing with its prevention and potential therapeutic interventions, especially during PCI. The role of gadolinium as an alternative to iodinated contrast is also discussed.

## 1. Introduction

There is a high prevalence of Chronic Kidney Disease (CKD) in Patients with Coronary Artery Disease (CAD), ranging from 23%–46% in different studies [1–3]. Patients with CKD also have a higher risk of cardiovascular events than the general population [4–6]. Also, cardiovascular disease accounts for more than half of end-stage renal disease (ESRD) deaths [7]. Reduced renal function has been seen to be independently associated with risk of death, cardiovascular events, and hospitalization in large, community-based populations [6, 8]. 

The incidence of renal insufficiency in this high-risk group of population undergoing coronary intervention is on the rise due to the use of radiographic contrast in increasingly complex cardiac interventional procedures [9]. This has a direct consequence on mortality as seen by Brown et al. who showed that a transient as well as persistent postprocedural renal dysfunction was prognostically significant for mortality during extended followup [10]. Renal function is directly related to mortality in patients with acute myocardial infarction undergoing Primary Percutaneous Coronary Intervention (PCI)-lower the Creatinine Clearance (CrCl); lesser is the survival after Primary PCI [11].

This paper covers the latest update on contrast-induced nephropathy among patients undergoing PCI.

## 2. Definition

Contrast-induced nephropathy (CIN) after PCI has multiple definitions in the medical literature [12, 13]. Due to these different definitions used across literature, existing data on this clinical entity is inconsistent. Harjai et al. attempted to identify the optimal definition of CIN by assessing the prognostic significance of 4 commonly used contemporary definitions of CIN (increases in serum creatinine after PCI [Cr] >1.0 mg/dL, >0.5 mg/dL, and >25% after PCI; and the American College of Cardiology National Cardiovascular Data Registry definition which defines it as a 2-fold increase in serum creatinine to >2.0 mg/dL or a need for dialysis after PCI [14]) with respect to 6-month major adverse cardiovascular events and all-cause mortality. Only 2 definitions ([Cr] ≥0.5 mg/dL, ≥25%) consistently predicted adverse events after PCI [15]. Therefore, a complete definition for CIN encompasses absolute (≥0.5 mg/dL) and relative increase (≥25%) in serum creatinine at 48–72 hours after exposure to a contrast agent compared to baseline serum creatinine values, when alternative explanations for renal impairment have been excluded [9].

## 3. Incidence of CIN

Based on these definitions, the overall incidence of CIN in the general population has been estimated to lie between 1%–6% [16]. The figure is higher when CIN follows PCI. In their retrospective analysis of the Mayo Clinic PCI registry comprising of 7586 patients, Rihal et al. found the incidence for general population undergoing PCI to be 3.3% and dialysis was needed in 0.3% [17]. However, incidence of CIN rises up to 20% or more in selected patient subsets, especially in patients with underlying cardiovascular disease [9].

## 4. Pathogenesis of CIN

Although the pathogenesis of CIN is not well understood, there is increasing evidence that it occurs as a combination of direct toxicity to the renal tubular epithelium, oxidative stress, ischemic injury, and renal tubular obstruction [18–20]. Also, increased intratubular pressure secondary to contrast-induced diuresis and increased perivascular hydrostatic pressure may lead to medullary hypoxia through lower medullary blood flow [21]. Renal ischemia may be the result of an imbalance between vasoactive substances (Adenosine and Endothelin) and vasodilators (NO and Prostaglandins) [19]. 

Significant urine volume is needed to clear the high osmotic load of the contrast medium. Exposure to this high osmotic load results in characteristic histopathological changes of osmotic nephrosis, a morphological pattern with vacuolization and swelling of the renal proximal tubular cells [22]. In a study, this finding was seen in almost a quarter (22%) of patients undergoing renal biopsy within 10 days of contrast exposure [23]. 

## 5. Risk Factors of CIN

The risk factors of CIN have been extensively studied in the past and can be classified into modifiable and nonmodifiable risk factors. These have also been subdivided into “patient related” and procedure related and are tabulated in Table 1. 

### 5.1. Age

The elderly remain at a higher risk of CIN after PCI, although the reason has not been elucidated yet. It is believed to be multifactorial, including age-related reduction in glomerular filtration rate (GFR), tubular function, as well as more difficult vascular access requiring greater amount of contrast, presence of multivessel disease, and comorbids. A few studies have found age older than 70 years to be an independent predictor of CIN in multivariate analysis [24–26].

### 5.2. Pre-existing Renal Disease

Baseline elevated serum creatinine was found to be an independent predictor of CIN in numerous studies. Increase in baseline Cr level to 1.2 mg/dL or higher resulted in an exponential increase in the risk of nephrotoxicity [27]. Risk of developing CIN after undergoing PCI rises with lowering GFR [28].

### 5.3. Diabetes Mellitus

Many studies have found Diabetes Mellitus (DM) as an independent risk factor for CIN in studies [17, 26, 29]. This risk is also independent of preexisting renal dysfunction [26, 30]. The incidence of CIN in diabetics varies from 5.7% to 29.4% [30, 31]. Given an increasing prevalence of DM in the general population and a higher risk of vascular disease among this group, diabetic patients represent a major fraction of patients requiring radiological procedures with use of contrast including PCI. Toprak et al. showed that in patients with preexisting renal insufficiency, Diabetes Mellitus independently increased the risk of development of CIN and need for dialysis as opposed to pre-DM and Normal Fasting states [32], while Berns showed that the incidence of CIN in diabetics was higher if Cr > 4.0 compared to Cr between 2.0 and 4.0 mg/dL. Clearly, there is a synergistic effect of diabetes and pre existing renal insufficiency [33].

### 5.4. Cardiac Risk Factors

Congestive Heart Failure, Anterior MI, Cardiogenic Shock, and Use of Intra-aortic Balloon Pump have all been associated with increased risk of CIN after PCI, mainly because they all lead to Reduced Renal Perfusion [17, 26, 34, 35].

### 5.5. Volume of Contrast

Volume of contrast remains the primary modifiable risk factor. Increasing complexity of coronary intervention invariably has led to higher volumes used per procedure and this overall augments the risk of CIN. Many studies have documented a clear correlation between volume of contrast and risk of CIN [24, 36–38]. There is also an association of CIN with closely placed procedures which ultimately translates into an increased amount of contrast used cumulatively [36]. McCullough et al. showed that the risk of CIN is minimal in patients receiving less than 100 mL of contrast media during procedures, or the volume of contrast used is <5 mL/kg/Serum Cr [29].

However, whether incidence of CIN is dose related or not has also been studied. In their study of 118 patients with Cr >1.3 and pre existent renal disease, Mekan et al. found that the contrast-induced reduction in renal function was not significantly higher with a higher volume of contrast (>100 mL) [39]. On the other hand, Kane et al. demonstrated a significant rise in incidence of CIN with increase of volume of contrast [38]. The ratio of the volume of contrast media to the creatinine clearance (V/CrCl) has been correlated with the area under the curve of contrast media concentration over time. With that concept, Laskey et al. showed that a V/CrCl ratio >3.7 was a significant and independent predictor of an early abnormal increase in serum creatinine after PCI in an unselected population of 3,179 patients [40].

### 5.6. Type of Contrast

Another important factor contributing to risk of CIN is the type of contrast used, with osmolality playing the key role. Other differences in contrast media including ionicity and viscosity may also be involved. Properties of contrast media are listed in Table 2.

A meta analysis of 31 trials looking at CIN and osmolarity of contrast used revealed that the incidence of CIN was significantly higher with high osmolar contrast use in patients with pre existing renal insufficiency. In patients without renal disease it was not significantly different [41]. This was reconfirmed by Rudnick et al. who compared low-osmolar nonionic contrast agent, iohexol, and the high-osmolar ionic contrast agent, diatrizoate, in 1,196 patients undergoing cardiac angiography. They found that acute contrast nephrotoxicity was significantly higher in patients receiving diatrizoate. Again, this difference was limited to patients with previous renal insufficiency or renal insufficiency combined with diabetes mellitus [42].

The advent of iso-osmolar contrast was further promising. In a randomized, double-blind, prospective, multicenter study, Aspelin et al. compared the nephrotoxic effects of an iso-osmolar, dimeric, nonionic contrast medium, iodixanol, with those of a low-osmolar, nonionic, monomeric contrast medium, iohexol in a group of 129 diabetic patients with serum creatinine concentrations of 1.5 to 3.5 mg per decilitre, and showed that nephropathy induced by contrast medium may be less likely to develop in high-risk patients when iodixanol is used rather than a low-osmolar, nonionic contrast medium [43]. Another meta-analysis of pooled data of 16 double-blind, randomized, controlled trials from 2727 patients comparing iso-osmolar contrast medium iodixanol with low-osmolar contrast media was conducted [44]. The maximum rise of Serum Creatinine and frequency of CIN were both significantly lower in patients using iso-osmolar contrast. This was seen in all patients: renal insufficiency patients and in patients with combination of renal insufficiency and diabetes mellitus. Independent predictors of CIN included CKD, DM + KD and use of low osmolar contrast media.

Two recent trials looked at the incidence of CIN in CKD patients with use of iso-osmolar and low-osmolar contrast agents [45]. In the RECOVER trial, the incidence of CIN was significantly lower with iodixanol (7.9%) than with ioxaglate (17.0%; P 1/4 0.021). However, the ICON trial showed no significant difference in incidence of CIN [46]. The controversy continued with the CARE [47] and the NEPHRIC trials with the former showing no difference in the incidence of contrast-induced nephropathy in CKD patients treated with iopamidol or iodixanol, and the latter revealing iodixanol to be superior to iohexol in patients with CKD and DM [48].

In the most recent multicenter, double-blind, randomized, parallel-group ACTIVE trials, 148 patients with moderate-to-severe chronic kidney disease, undergoing contrast-enhanced multidetector computed tomography of the liver, were randomized to either the low-osmolar agent iomeprol-400 or iodixanol-320. The incidence of CIN as well as mean rise in serum creatinine was significantly higher in the patient group receiving iodixanol [49]. 

Generally the use of iso-osmolar contrast agents is safer and leads to lower rates of CIN in patients at high risk of developing acute kidney damage. However, it may not be needed in all patients. An expert consensus on this issue is lacking. Furthermore, the iso-osmolar media generally have higher viscosity than their low-osmolar monomeric counterparts. Hence, these media should be prewarmed before infusion, which markedly lowers the viscosity [50].

## 6. Risk Assessment and Scoring Systems

Patients usually have a combination of risk factors predisposing them to CIN after PCI. All scoring systems, therefore, attempt to encompass these risk factors. Bartholomew et al. worked on developing a time insensitive scoring system [51]. Independent variables (with weighted scores) included estimated creatinine clearance <60 mL/min (2), urgent PCI (2), intra-aortic balloon pump use (2), diabetes mellitus (1), congestive heart failure (1), hypertension (1), peripheral vascular disease (1), and contrast volume >260 mL (1). Incidence of CIN in their study was 2% [51].

Mehran et al., similarly, found eight variables (hypotension, intra-aortic balloon pump, congestive heart failure, chronic kidney disease, diabetes, age >75 years, anemia, and volume of contrast) and assigned a weighted integer to each variable to make up a score cumulatively so as to divide low risk (≥5) and high risk (≥16) scores [35]. The overall occurrence of CIN in the development set was 13.1%. Table 3 shows their algorithm.

## 7. Prognosis

CIN is the leading cause of acute renal failure in hospitalized patients and associated with prolonged in-hospital stay and increased morbidity, mortality, and costs. A considerable fraction of this in-hospital development of ARF has been due to the use of contrast media in radiographic procedures [29, 52]. In their retrospective analysis of the Mayo Clinic PCI registry, Rihal et al. found that 22% of the patients with ARF died during the index hospitalization compared with only 1.4% of patients without ARF (*P* < .0001). One-year and 5-year estimated mortality rates were also significantly higher in patients with ARF [17]. Mortality ramped up not only with ARF but even more if dialysis was needed as shown by McCullough et al. [29].

A retrospective analysis of 16,248 patients exposed to contrast media showed that even apparently small decreases in renal function can lead to excessive mortality rates independent of other risk factors, and given that small rises in serum creatinine levels actually represent a significant drop in GFR [53]. Furthermore, renal insufficiency has been found to be a strong independent predictor of death and subsequent cardiac events in a dose-dependent fashion during and after PCI [54].

## 8. Prevention of Radiocontrast Nephropathy

### 8.1. Volume Administration

Volume administration remains the key factor for the prevention of CIN, even though no randomized controlled trial has compared a strategy of volume expansion with no volume expansion has been performed. Possible mechanisms for prevention of renal insult by fluid administration include plasma volume expansion with concomitant suppression of the rennin-angiotensin-aldosterone system with the increased delivery of Na to distal nephron, downregulation of the tubuloglomerular feedback, dilution of the CM, and thus prevention of renal cortical vasoconstriction—and avoidance of tubular obstruction [55]. It may also be due to reduction in Nitric Oxide production.

Several trials have assessed the optimum type, amount, duration, and route of fluid administration to prevent CIN [56–59]. However, much of these aspects remain unclear. 

Trivedi et al. found that oral fluid administration alone appeared to be inferior to intravenous volume expansion with respect to the development of CIN [59]. Solomon et al. demonstrated the superiority of intravenous 0.45% Saline administration (starting 4–6 hours preprocedure and continued for 24 hours postprocedure) over IV Saline plus Furosemide and IV Saline and Mannitol in patients with mild renal insufficiency [56]. 

In a study conducted by Stevens et al., 98 participants were randomized to forced diuresis with intravenous crystalloid, furosemide, mannitol (if pulmonary capillary wedge pressure <20 mm Hg), and low-dose dopamine (*n* = 43) versus intravenous crystalloid and matching placebos (*n* = 55). They showed that Plain IV Fluid administration is as good as IV Fluid administration + Dopamine + Lasix + Mannitol as long as urine flow rates greater than 150 mL/h in the postprocedure period were achieved [57].

Recently, Mueller et al. showed that volume expansion with isotonic saline is superior to half-isotonic saline, possibly explained by its enhanced ability to expand intravascular volume [60]. However, since this study was conducted on low risk patients with normal renal function, these results cannot be transferred conclusively to patients with moderate and severe chronic renal failure. 

The CIN Consensus Working Panel published strategies to reduced contrast nephropathy a few years ago. Adequate intravenous volume expansion with isotonic crystalloid (1.0–1.5 mL/kg/hr) for 3–12 hours before the procedure and continued for 6 to 24 hours to prevent development of CIN in patients at risk. Caution is needed in patients with CHF. They can benefit from optimal hemodynamic stabilization than excessive volume administration [61]. This was supported by two further studies [62, 63].

Merten et al. found that volume expansion with sodium bicarbonate (154 mEq/L of sodium bicarbonate in dextrose and water at a rate of 3 mL/kg/hour per 1 hour before CM exposure, followed by 1 mL/kg/hour during, and for 6 hours after the procedure) was more effective than with sodium chloride for prophylaxis of contrast-induced renal failure and may provide additional renoprotection by alkalinizing renal tubular fluid, which the authors hypothesized would decrease tubular damage by scavenging oxygen free radicals [64]. Over the years, a number of randomized controlled trials comparing volume administration regimens using sodium bicarbonate and sodium chloride have given conflicting results, [65–69] with systematic reviews and meta analyses performed on these trials inconsistently reporting superiority of sodium bicarbonate over sodium chloride [70–72]. This may be due to heterogeneity between trials and the large number of unpublished studies. Also, Zoungas et al., in their systematic review found that smaller studies measuring outcome relatively early were prone to report superiority of sodium bicarbonate whereas larger studies provided more neutral results between the two fluid administration regimens [72]. 

Finally, Clavijo et al. conducted a retrospective analysis and demonstrated that a rapid intraarterial infusion of dextrose 5% (1 L administered through the femoral artery sheath as a bolus >5 minutes immediately before angiography) was well tolerated and effective against CIN in patients with a creatinine clearance ≤60 mL/min [73]. 

Although, the key is adequate volume expansion for the prevention of CIN, the prognostic implication of this strategy, however, is missing. There is also a lack in sufficient data to support a single best volume administration strategy (optimal timing, type, volume, and rate of fluid administration). Current evidence remains uncertain about any superiority of sodium bicarbonate over sodium chloride in preventing the development of CIN. Also, special emphasis is needed on patients with heart failure and preexisting renal failure as these patients are high risk for CIN and have been poorly represented in existing studies on volume administration. Also, these patients will be more challenging in subjecting to high volume expansion.

### 8.2. Acetylcysteine

So far, N-acetylcysteine (NAC) has been the most widely studied of all prophylaxis strategies. Several possible mechanisms exist by which this drug protects renal tissue from CM. One such mechanism is by direct vasodilation of kidney vessels, contributing to improved renal hemodynamics [74]. It may also decrease endothelial dysfunction, and, most importantly, it is able to scavenge oxygen free radicals, thus preventing direct oxidative tissue damage occurring in patients receiving CM. This property of NAC as an antioxidant gave it a popularity as a promising drug for prevention of CIN. 

In a landmark randomized placebo controlled trial conducted by Tepel et al., it was first seen that prophylactic oral administration of the antioxidant acetylcysteine, along with fluid administration, prevented the reduction in renal function induced by contrast agents in patients with chronic renal insufficiency [75]. They showed that the incidence of CIN as well as the decrease in mean serum creatinine concentration 48 hours after administration of CM was significantly less in the acetylcysteine group. This benefit was confirmed by the Acetylcysteine to Prevent Angiography-Related Renal Tissue Injury (APART) trial, which showed that NAC reduced the risk of postcardiac catheterization nephropathy in patients with chronic renal insufficiency and decreased ejection fraction [76]. Thus, it was considered as routine prophylaxis in patients with chronic renal insufficiency undergoing cardiac catheterization. 

However, recently, there has been a declining enthusiasm for the efficacy of NAC as numerous studies have failed to show a significant benefit of acetylcysteine compared to controls (Table 4) [77–82]. 

Researchers then investigated whether increasing the standard dose of acetylcysteine (600 mg orally twice daily) resulted in better renoprotection. Hence, Briguori et al., in their single center study, found that double dose of NAC (1200 mg orally twice daily) resulted in better prevention of CIN, especially with high volumes (140 mL) of nonionic, low-osmolality contrast agent [83]. Efficacy of IV acetylcysteine was investigated by Baker et al. in their RAPPID trial [84]. They randomised 80 patients with stable renal dysfunction undergoing cardiac catheterization/intervention to a rapid protocol of IV NAC (150 mg/kg over 30 minutes immediately before contrast followed by 50 mg/kg over 4 hours) or IV fluid administration (1 mL/kg/h N/saline for 12 hours pre- and postcontrast) and followed them prospectively for development of Radiocontrast-induced nephropathy. Even though NAC infusion was ceased after the bolus in three patients (7%) due to flushing, itching, or a transient rash, overall, the trial showed a significantly lower incidence of CIN in treated patients as compared with controls (5% versus 21%; P 1/4 0.04). Therefore, IV NAC emerged as a reasonable option in all patients at risk of CIN before contrast exposure when time constraints preclude adequate oral prophylaxis, provided that the patient is able to tolerate this degree of volume loading [84].

A meta-analysis of twenty trials involving 2195 patients showed summary risk ratio for contrast-related nephropathy to be 0.73 (95% confidence interval: 0.52 to 1.0; *P* = .08), a nonsignificant trend towards benefit in patients treated with acetylcysteine [85]. Meta-analyses generally conclude that there may be some benefit of NAC in reducing CIN [85, 86]. These trials were, however, very heterogeneous with inconsistent results. High-quality, large clinical trials that are needed before acetylcysteine use in this indication can be recommended universally.

Moreover, recent studies have assessed combined efficacy of two most important preventive interventions, use of NAC and bicarbonate [62, 63]. These included the REMEDIAL trial that showed the strategy of volume supplementation by sodium bicarbonate plus NAC to be superior to the combination of normal saline with NAC alone or with the addition of ascorbic acid in preventing CIN in patients at medium-to-high risk [62]. However, Maioli et al. had contradicting results in their prospective single center study including 502 patients with renal dysfunction with no advantage of NAC and Sodium Bicarbonate to NAC and Saline administration [87].

### 8.3. Statins

Statins have also been studied as potential agents to prevent CIN in patients undergoing PCI, primarily by means of their beneficial effects on endothelial function and oxidative stress. This is very promising as most patients undergoing PCI are already on this class of drugs. Patti et al. showed that Statin-treated patients had a significantly lower incidence of CIN (3% versus 27%, *P* < .0001; 90% risk decrease) and had better postprocedural creatinine clearance (80 ± 20 versus 65 ± 16 mL/min, *P* < .0001). Benefit of statin before treatment was observed in all subgroups, except in patients with a preexisting creatinine clearance <40 mL/min [88]. Similar results were obtained in two other large retrospective studies [89, 90]. However, in the prospective, randomized, double-blind, placebo-controlled, 2-center PROMISS trial, involving 247 consecutive patients, Simvastatin pretreatment for short term at high dose did not prevent renal function deterioration after administration of contrast medium in patients with baseline renal insufficiency undergoing coronary angiography. Another prospective, randomized, placebo control trial, involving 304 patients with baseline Chronic Kidney Disease, did not demonstrate any additional benefit of 80 mg Atorvastatin pretreatment for the prevention of CIN in these patients [91]. However, a prospective randomized trial showed favourable results while using high dose Simvastatin (80 mg) pretreatment instead of low dose (20 mg) to prevent CIN [92]. Overall, strong evidence is not present to support the use of statins, especially in patients where it is not otherwise indicated.

### 8.4. Other Interventions

Numerous other interventions have been studied to assess their efficacy in preventing CIN. Overall, results have either been ambiguous or unsatisfactory. A list of these potential management strategies and the existing data is summarized in Table 5.

## 9. Gadolinium

Gadolinium is often used as an alternative to iodinated contrast in patients at increased risk of CIN. Overall, the trials show conflicting results about whether gadolinium is better than iodinated contrast in its risk of CIN (Table 6). The safety of gadolinium in patients at increased risk of CIN has not been established yet. In their systemic review of 17 studies, Boyden et al. reported both favourable and negative results with regard to the association of gadolinium and CIN. The differences in the results appeared to be dose related. When gadolinium was used in doses of 0.4 mmol/kg or higher, there appeared to be an increased incidence of ARF particularly in patients with preexisting renal insufficiency [116]. Furthermore, the risk of precipitating nephrogenic systemic fibrosis (NSF) through administration of Gadolinium in patients already renally compromised must be emphasized [117]. NSF seems to be associated with administration of higher than usual doses of Gadolinium, according to one study [118]. Hence, till further evidence becomes available, administration of Gadolinium to decrease CIN incidence appears questionable, especially in the light of its particular adverse effects.

## 10. Conclusion

CIN is an iatrogenic disorder with high morbidity and mortality and a high incidence in elderly, diabetics, and patients with pre existing renal failure. Despite uncertainty regarding the degree of nephrotoxicity produced by various contrast agents, nonionic low-osmolar or iso-osmolar contrast media remains the preferred choice. Limiting the volume of contrast as much as possible is recommended. Best way to prevent CIN is to identify patients at high risk and provide adequate volume administration. However, use of sodium bicarbonate or N-Acetylcysteine in its prevention remains inconclusive in light of available evidence. Although the role of various drugs in prevention remains controversial, nephrotoxic drugs should be avoided before and after the procedure. Also, there still is no conclusive evidence to recommend gadolinium as a better alternative to iodinated contrast media in order to prevent CIN.

##  Conflict of Interests

The authors declare that they have no conflict of interests.

## Figures and Tables

**Table 1:** Risk factors for development of contrast-induced nephropathy.

Nonmodifiable	Modifiable
Patient-related factors	
Age	
Diabetes mellitus with CRF	
Preexisting renal failure	Volume depletion
Congestive heart failure	Anemia, PCI related blood loss
Hemodynamic instability	Nephrotoxic drug use
Nephrotic syndrome	Low serum albumin
Renal transplant	

Procedure-related factors	

IABP	Volume of contrast media
Emergent/primary PCI	Multiple administration of CM within 72 hours
Intraarterial CM administration	Osmolality and ionicity of CM

CM, contrast media; CRF, chronic renal failure; IABP, intra-aortic balloon pump; PCI, percutaneous coronary intervention.

**Table 2:** Properties of contrast media.

Generic name	Osmolarity	Ionicity	Viscosity (mPa.s at 20 C)
Diatrizoate			n/a^§^
Iothalamate	High-osmolar	Ionic monomer	n/a^§^
Ioxithalamate			26.0
Ioxaglate		Ionic dimer	15.7
Iohexol			20.4
Iopamidol	Low-osmolar		20.9
Ioversol		Nonionic monomer	18.0
Iopromide			22.0
Iobitridol			n/a^§^
Iomeprol			n/a^§^
Iodixanol	Iso-osmolar	Nonionic dimer	26.6

^§^Not Available.

**Table:** (a)

Risk factors	Score
Hypotension	5
IABP	5
CHF	5
Age>75	4
Anemia	3
Diabetes	3
Contrast volume	1/100 cc
Serum Cr> 1.5	4
OR	
GFR<60 mL/min/1.73 m2	2 for 40–60
	4 for 20–40
	6 for <20

**Table:** (b)

Risk score	Risk of CIN	Risk of dialysis
<5	7.5%	0.04%
6–10	14%	0.12%
11–16	26.1%	1.09%
>16	57.3%	12.6%

**Table 4:** Studies comparing acetylcysteine administration to control arm in patients undergoing coronary angiography.

Study (N)	CIN definition	Acetylcysteine	Control	*P* value
Azmus [77] (397)	≥25% or ≥0.5 mg/dL, increase in SCr at 48 hours	7.1	8.4	.62
Boccalandro [81] **(**179)	≥0.5 mg/dL increase in SCr at 48 hours	13	12	.84
Briguori [82] (183)	≥25% increase in Scr at 48 hours	65	11	.22
Kay [79] (200)	≥25% increase in SCr at 48 hours	4	12	.03
Marenzi [78] (354)	≥25% increase in SCr at 72 hours	15 versus 8^a^	30	<.001
Webb [80] (487)	>5 mL/min decrease in CrCl	23.3	20.7	.57

CIN, contrast-induced nephropathy; CrCl. Creatinine clearance; IV, intravenous; SCr, serum creatinine, dd, double dose

^a^Three arm study: standard dose versus high dose versus placebo.

**Table 5:** List of agents assessed for prevention of CIN and the literature available on each.

Agent	Mechanism	Comment
Dopamine [93–95]	Renal vasodilatation	No clear benefit
		Deleterious in PVD
Fenoldopam [96–98]	Selective dopa 1 receptor agonist	No clear benefit
		May be intrarenal at higher doses
Theophylline [95, 99–101]	Adenosine receptor antagonist	No clear benefit
Calcium Channel Blockers [102–104]	Relieve vasoconstriction	No clear benefit
Prostaglandin E [105]	Vasodilatation	May be beneficial
Ascorbic Acid [106]	Antioxidant	May be beneficial
ANP [107]	Vasodilatation	No benefit
Hemodialysis [108, 109]	Removal of offending agent	No benefit
Hemofiltration [110, 111]	Continuous	May be beneficial

**Table 6:** Studies assessing incidence of CIN in patients with exposure of gadolinium as contrast.

Study	Sample design	Contrast dose	Renal status	CIN definition	Renal outcome	Preventive treatment used
Spinosa (2000) [112]	35-Randomized	0.29 mmol/kg (0.13–0.40 mmol/kg) Gadolinium; 51 mL (33–80 mL) nonionic contrast	Cr > 1.5 mg/dL	>0.5 mg/dL increase in [Cr ] within 48 hours	5 % (1/20) exposed to Gadolinium; 40% (6/15) exposed to nonionic contrast	Volume administration

Erley (2004) [113]	21-Prospective, randomized study	0.57 ± 0.17 mmol/kg Gadolinium; 0.60 ± 0.271 mmol/kg Iohexol	Cr > 1.5 mg/dL or GFR < 50 mL/min	>50% decrease in GFR	50% (5/10) exposed to Gadolinium and 45% (5/11) exposed to Iohexol	Volume administration

Briguori (2006) [114]	57-Retrospective study	0.6 ± 0.3 mmol/kg Gadolinium based; 122 ± 58 mL Iodixanol	Cr > 2 mg/dL or CrCL < 40 mL/min	>0.5 mg/dL increase in [Cr ] within 48 hours or need for dialysis within 5 days	28% (7/ 25) exposed to Gadolinium plus iodinated contrast and 6.5% (2/32) exposed to iodinated contrast alone	Normal saline plus N-acetylcysteine

Reed (2007) [115]	169-Retrospective Study	151 ± 79 mL Gadolinium (with Iodinated contrast dilution); 136 ± 72 mL iodinated contrast	CrCl < 60 mL/min/1.73 m^2^, a serum Cr level >1.5 mL/dL, and not on hemodialysis.	≥0.5 mg/dL increase in [Cr] within 5 days	16% (14/90) of those exposed to diluted Gadolinium and 14% (11/79) of Iodinated contrast	N-acetylcysteine plus Volume administration

Kane [38] (2008)	163-Retrospective Study	3 comparative groups receiving 76 ± 40 mL Gadolinium; 55 ± 35 Gadolinium (with 37 ± 37 Iodinated contrast); 102 ± 50 Iodinated contrast	Cr ≥ 2 mg/dL	≥0.5 mg/dL increase in [Cr] within 7 days	5.3% (3/57) in Gadolinium only; 10.5% (4/38) in Gadolinium + Iodinated Contrast; 20.6% (14/68) in Iodinated contrast alone	N-acetylcysteine plus Volume administration

CrCL: Creatinine Clearance; Cr: Serum Creatinine GFR: Glomerular Filtration Rate.
